# Consumption of *Bt* Maize Pollen Containing Cry1Ie Does Not Negatively Affect *Propylea japonica* (Thunberg) (Coleoptera: Coccinellidae)

**DOI:** 10.3390/toxins9030108

**Published:** 2017-03-16

**Authors:** Yonghui Li, Yanmin Liu, Xinming Yin, Jörg Romeis, Xinyuan Song, Xiuping Chen, Lili Geng, Yufa Peng, Yunhe Li

**Affiliations:** 1 College of Plant Protection, Henan Agricultural University, Zhengzhou 450002, China; liyonghuind@126.com; 2 State Key Laboratory for Biology of Plant Diseases and Insect Pests, Institute of Plant Protection, Chinese Academy of Agricultural Sciences, Beijing 100193, China; liuyanmin2017@163.com (Y.L.); joerg.romeis@agroscope.admin.ch (J.R.); xpchen@ippcaas.cn (X.C.); llgeng@ippcaas.cn (L.G.); yfpeng@ippcaas.cn (Y.P.); 3 Agroscope, Biosafety Research Group, 8046 Zurich, Switzerland; 4 Jilin Academy of Agricultural Sciences, Changchun, Jilin 130124, China; songxinyuan1980@163.com

**Keywords:** *Bt* maize, Cry1Ie, non-target effects, ladybirds, environmental risk assessment

## Abstract

*Propylea japonica* (Thunberg) (Coleoptera: Coccinellidae) are prevalent predators and pollen feeders in East Asian maize fields. They are therefore indirectly (via prey) and directly (via pollen) exposed to Cry proteins within *Bt*-transgenic maize fields. The effects of Cry1Ie-producing transgenic maize pollen on the fitness of *P. japonica* was assessed using two dietary-exposure experiments in the laboratory. In the first experiment, survival, larval developmental time, adult fresh weight, and fecundity did not differ between ladybirds consuming *Bt* or non-*Bt* maize pollen. In the second experiment, none of the tested lethal and sublethal parameters of *P. japonica* were negatively affected when fed a rapeseed pollen-based diet containing Cry1Ie protein at 200 μg/g dry weight of diet. In contrast, the larval developmental time, adult fresh weight, and fecundity of *P. japonica* were significantly adversely affected when fed diet containing the positive control compound E-64. In both experiments, the bioactivity of the Cry1Ie protein in the food sources was confirmed by bioassays with a Cry1Ie-sensitive lepidopteran species. These results indicated that *P. japonica* are not affected by the consumption of Cry1Ie-expressing maize pollen and are not sensitive to the Cry1Ie protein, suggesting that the growing of *Bt* maize expressing Cry1Ie protein will pose a negligible risk to *P. japonica*.

## 1. Introduction

Maize (*Zea mays* L.) is one of the most important cereal crops in the world and plays a decisive role in food and feed production. In China, it is mainly grown for food, feed, and ethanol production [1]. However, the yield of maize can be reduced heavily by insect pests. The most important maize pest in China is the Asia maize borer, *Ostrinia furnacalis* Guenée (Lepidoptera: Crambidae). The Asia maize borer was estimated to cause approximately 10% of yield loss each year and more than 30% in years of heavy infestations [2].

Insect-resistant genetically engineered (IRGE) crops expressing insecticidal proteins derived from the soil bacterium *Bacillus thuringiensis* (*Bt*) provide a powerful and environmentally friendly strategy for insect pest control. Since their first commercialization in 1996, the adoption of *Bt*-transgenic cotton and maize varieties increased steadily, reaching 179 million hectares in 28 countries in 2015, with *Bt* maize been grown on 53.9 million hectares in 19 countries [3].

China has devoted great efforts to develop *Bt* maize. To date, China has obtained many *Bt* maize lines, all of which express *cry1* and/or *cry2* genes targeting lepidopteran pests [4,5,6,7]. Most of the *Bt* maize lines, such as IE09S034 (expressing a *cry1Ie* gene), BT-799 (expressing a modified *mcry1Ac* gene), and Shuangkang12-5 (expressing a fusion *cry1Ab/2Aj* gene) have proven to be effective against lepidopteran pests [8,9]. In the case of *Bt* maize expressing *cry1Ie*, the Chinese Ministry of Agriculture has already approved field trials for assessing the environmental risks, indicating its potential to be commercialized in the near future.

Prior to the commercial release of IRGE crops, it is crucial to evaluate their potential effects on the environment, in particular on non-target organisms that fulfill important ecological functions [10,11,12]. This includes organisms that contribute to the biological control of pests [13,14,15], pollination [16], or decomposition [17,18]. The ladybird, *Propylea japonica* (Thunberg) (Coleoptera: Coccinellidae), is an important predator in many crop systems throughout East Asia [18]. Both larvae and adults are predaceous, feeding predominantly on aphids, planthoppers, and the eggs and young larvae of lepidopterans [19,20]. During plant anthesis, they will also use plant pollen as a supplemental food source when insect prey is scarce [21,22]. Thus, once *Bt* maize is commercially grown in China, the ladybird has the potential to be exposed to plant-produced insecticidal proteins not merely by feeding on herbivores but also by directly feeding on *Bt* maize pollen.

In the current study, we investigated the potential dietary effects of *Bt* maize pollen containing Cry1Ie on *P. japonica*. In addition, a second experiment was conducted in which the ladybirds were directly exposed to purified Cry1Ie protein mixed in an established rapeseed pollen-based diet.

## 2. Results

### 2.1. Bt Maize Pollen Experiment

#### 2.1.1. Effects on Life Table Parameters

When fed with maize pollen, over 73% of the *P. japonica* larvae developed to adults, and the pupation rates and eclosion rates did not significantly differ between the *Bt* and the non-*Bt* maize pollen treatments (both *P* > 0.05) (Table 1). Larval development time (days to pupa) and adult fresh weight were not significantly affected, either, by feeding on *Bt* pollen (development time: *U* = 2008.5, *P* = 0.63; *t =* 0.38 *df* = 61; adult weight: *P* = 0.71 for females and *t =* −1.09, *df* = 49, *P* = 0.28 for males). Similarly, the fecundity of *P. japonica* females was not significantly affected by feeding on *Bt* pollen (*t =* −0.93, *df* = 43, *P* = 0.36) (Table 1).

#### 2.1.2. Bioactivity of Cry1Ie Protein in Maize Pollen

The mean (±SE) weight of the Cry1Ie-sensitive *Chilo suppressalis* Walker (Lepidoptera: Crambidae) larvae was significantly reduced when fed on an artificial diet containing fresh *Bt* maize pollen for seven days (0.22 ± 0.01 mg) compared to larvae fed on fresh control maize pollen (4.14 ± 0.29 mg) (*t* = 13.6, *df* = 57, *P* ˂ 0.001). *C. suppressalis* larval weight was slightly, but significantly, higher when fed *Bt* maize pollen that had been exposed to *P. japonica* for two days (0.26 ± 0.01 mg) as compared to freshly prepared *Bt* maize pollen (0.22 ± 0.01 mg) (*t* = −2.12, *df* = 57, *P* = 0.038).

### 2.2. Purified Cry Protein Experiment

#### 2.2.1. Effects on Life Table Parameters

Pair-wise comparisons revealed that the treatment containing Cry1Ie protein did not differ significantly from the untreated (negative) control for any of the *P. japonica* test parameters including pupation rate, ecolosion rate, development time, adult fresh weight (male/female), and total fecundity (total number of eggs laid per female) (all *P* > 0.05) (Table 2). In contrast, the larval development time of *P. japonica* was significantly prolonged (*U* = 35.0, *P* < 0.001), and the mean weight of the freshly emerged adults was significantly reduced when feeding on diet containing E-64 (Dunnett test; *P* < 0.001 for females, and *P* = 0.002 for males). No significant difference was detected for pupation rate, eclosion rate, and the survival rate of *P. japonica* between the untreated control treatment and the E-64 treatment (all *P* > 0.05) (Table 2).

#### 2.2.2. Bioactivity of Cry1Ie Protein in Rapeseed Pollen

Sensitive-insect bioassays indicated that the mean (±SE) weight of *C. suppressalis* larvae was significantly reduced when fed an artificial diet containing Cry1Ie (0.26 ± 0.02 mg) for seven days compared to those fed untreated control diet (0.50 ± 0.06 mg) (*t* = 4.02, *df* = 52, *P* ˂ 0.001). No statistical differences was detected for the mean weight of *C. suppressalis* larvae when fed a Cry1Ie-containing diet that had been freshly prepared (0.26 ± 0.02 mg) compared to a diet that had been exposed to *P. japonica* for two days (0.28 ± 0.02 mg) (*t* = −0.58, *df* = 55, *P* = 0.57).

## 3. Discussion

The *cry1Ie* gene has been identified from *Bacillus thuringiensis* isolate Btc007 and is a relatively new gene used for plant transformation [23]. It was found that a transgenic maize line expressing *cry1Ie* was highly resistant against the stem borer *O. furnacalis* [24]. In addition, it appears that the Cry1Ie protein has no cross-resistance with other Lepidoptera-active insecticidal proteins such as Cry1Ab, Cry1Ac, Cry1Ah, or Cry1F [25,26,27], making it a suitable candidate gene for developing stacked events for improved pest control. However, our knowledge regarding the potential effects of the Cry1Ie protein on non-target beneficial arthropods is still limited.

Our bioassays revealed no adverse effects of *Bt* maize pollen containing Cry1Ie on the fitness of *P. japonica*. In our feeding experiment, the ladybirds were continually fed on *Bt* maize pollen for more than four weeks, while the pollen shedding period of maize normally lasts for 5–8 days with a maximum of 14 days [28,29]. In addition, our previous study had confirmed that *P. japonica* ingested much larger amounts of maize pollen under laboratory, confined conditions when compared to the field situation [30]. Consequently, the ladybirds in our laboratory bioassays were exposed to Cry1Ie protein at much higher levels than in the field. In addition, the bioactivity of Cry1Ie in the pollen samples used in the experiments was confirmed by sensitive insect bioassays using larvae of *C. suppressalis*. This demonstrates that *P. japonica* was exposed to a constant and elevated level of active Cry1Ie protein.

To further confirm that *P. japonica* is not sensitive to Cry1Ie, a second experiment was conducted where the ladybirds were directly fed purified Cry1Ie protein at a very high dose of 200 μg/g dry weight of diet using a validated test system [31]. No detrimental effects were detected on the tested lethal and sublethal life table parameters. The positive control (E-64) treatment, in contrast, caused a significant prolongation of the larval development time, a lower adult fresh weight, and a decrease in fecundity. The results demonstrate that *P. japonica* indeed ingested Cry1Ie protein and that the experimental system used in the current study was able to detect adverse effects, if present. Furthermore, the bioactivity of Cry1Ie protein was confirmed by sensitive insect bioassays with the same batch of Cry protein, suggesting that the test insects were exposed to bioactive Cry1Ie protein during the duration of the bioassay. In addition, the Cry1Ie protein concentration in this experiment was approximately 40 times of the concentration in the Cry1Ie-transgenic maize pollen (5 μg/g fresh weight) [32]. Therefore, we can conclude that *P. japonica* is not sensitive to Cry1Ie at a level much higher than they may encounter in *Bt* maize fields.

Because of the ecological importance as a natural enemy and its availability and amenability for laboratory studies, *P. japonica* has been selected as representative species to support the risk assessment of IRGE crops [33]. Our previous study indicated that *P. japonica* larvae are not sensitive to Cry1Ab, Cry1Ac, and Cry1F proteins [31], which are produced in different *Bt*-transgenic crops, including maize, cotton, and rice. In addition, feeding experiments with *Bt* rice pollen showed that the fitness of *P. japonica* was not adversely affected by consumption of pollen containing Cry1Ab, Cry1C, or Cry2A [34,35]. Similarly, ingestion of *Bt* maize pollen containing Cry1Ab/2Aj or Cry1Ac had no detrimental effect on *P. japonica* larvae [30]. Although a previous study reported that consumption of Cry1Ah-containing maize pollen affected the activity of some gut enzymes of *P. japonica* [36], no effect was detected on the growth or development of *P. japonica* larvae when fed Cry1Ah-containing maize pollen [37]. Similarly, a tritrophic studies showed that the development of *P. japonica* was not affected when fed with *Nilaparvata lugens* (Stål) (Hemiptera: Delphacidae) that had been reared on Cry1Ab-contained *Bt* rice [38]. The current data complement our knowledge on the toxicity of Cry proteins to *P. japonica*. Overall, the available results demonstrate that this important predatory ladybird species is not sensitive to Cry proteins that are widely used for plant transformation to control lepidopteran pests or might be used in the future.

To our knowledge, the current study is the first to assess the potential effects of *Bt* maize producing Cry1Ie protein on a predatory insect in the laboratory. So far, the non-target insect assessment concerning Cry1Ie has only focused on the survival of Chinese honey bees, *Aphis cerana cerana* (Hymenoptera: Apidae) [39], the diversity of the midgut bacteria of the worker bees, *Apis mellifera ligustica* (Hymenoptera: Apidae) [40], and the survival, pollen consumption, and olfactory learning of young adult honey bees, *A. mellifera* [41]. None of those studies has revealed any adverse effects of Cry1Ie consumption. In addition, two studies reported that Cry1Ie-expressing maize had no significant effects on the arthropod community in maize fields [42,43].

In summary, the present study shows that the consumption of *Bt* maize pollen expressing Cry1Ie does not negatively affect the fitness of *P. japonica* and that the ladybirds are not sensitive to Cry1Ie at 200 μg/g diet that is significantly higher than they may encounter in the *Bt* maize fields. Therefore, we conclude that growing of Cry1Ie expressing maize should pose a negligible risk to *P. japonica.*

## 4. Materials and Methods

### 4.1. Insects

Specimens of *P. japonica* were collected from an experiment maize field of the Institute of Plant Protection, Chinese Academy of Agricultural Sciences (CAAS), near Langfang City, Hebei Province, China (39.5° N, 116.7° E) in 2015. A colony was subsequently maintained in the laboratory without introduction of field-collected insects for over two generations. Both larvae and adults of *P. japonica* were reared on soybean seedlings infested with *Aphis glycines* Matsumura (Hemiptera: Aphididae). The aphids were replaced daily, ensuring *ad libitum* food for the developing *P. japonica*. Newly hatched *P. japonica* larvae (<12 h after emergence) were used for the experiments. A *Bt*-susceptible strain of *C. suppressalis* was maintained on an artificial diet for over 80 generations in the laboratory [44]. All insects were reared in a climatic chamber at 26 ± 1 °C, 75% ± 5% RH and a 16:8 h light: dark photoperiod.

### 4.2. Maize Plants and Pollen Collection

The transgenic maize line IE09S034 and the corresponding non-transformed near isoline, Z31 (Zong31), were used in the experiment. IE09S034 plants express a *cry1Ie* gene under the control of the maize ubiquitin promoter. The seeds of IE09S034 and Z31 were provided by the Institute of Crop Sciences, CAAS.

The maize lines were simultaneously planted in six adjacent plots (three plots per maize line) at the experimental field station of Jilin Academy of Agriculture Sciences in Gongzhuling City, Jilin Province, China (43°19’ N, 124°29’ E) in 2014. Each plot was approximately 0.04 ha. The maize seeds were sown on 25 May 2014. The plants were cultivated according to the common local agricultural practices but without insecticide sprays during the growing period.

During maize anthesis in late July 2014, maize pollen was collected daily by shaking the maize tassels in a plastic bag. The collected pollen was air dried at room temperature for 48 h and subsequently passed through a sieve with a mesh size of 0.2 mm to remove anthers and contaminants. Pollen collected from each maize line was pooled and stored at −60 °C until further use.

### 4.3. Insecticidal Compounds and Bee-Collected Pollen

Insecticidal compounds used in this study included the protease inhibitor E-64 [*N*-[*N*-(l-3-trans-carboxyoxirane-2-carbony1)-l-leucyl]-agmatine)] and the *Bt* protein Cry1Ie. E-64 was purchase from Sigma-Aldrich (St. Louis, MO, USA) with the purity of 95%. For production of Cry1Ie protein, the *cry1Ie* gene was subcloned into vector pET-21b and expressed in Escherichia coli BL21 (DE3). The recombinant strain was induced by 0.1 mM IPTG at 18 °C for 12 h. The soluble Cry1Ie protein in the supernatant was purified by Ni-NTA (QIAGEN, Dusseldorf, Germany) and eluted by 250 mM imidazole (50 mM Na_2_CO_3_, pH 10.5). The protein preparation and purity were examined by SDS-polyacrylamide gel electrophoresis (SDS-PAGE) using Image J software. Protein concentrations were determined using Pierce BCA protein assay kit (Thermo Scientific, Waltham, MA, USA). The concentration of Cry1Ie protein in sodium carbonate solution (50 mM) was 200 μg/mL.

Bee-collected rapeseed pollen used in the experiments was purchased from China-Bee Science & Technology Development Co., Ltd (Beijing, China).

### 4.4. Feeding System for *P. japonica*

The pollen-based diet used in present study was developed and validated in previous studies and has been successfully used to assess the potential effects of *Bt* rice and *Bt* maize pollen or purified Cry proteins (mixed into a rapeseed pollen-based diet) on *P. japonica* [30,31,35]. The *P. japonica* larvae were individually confined in Petri dishes (6.0 cm diameter, 1.5 cm height). Larvae were fed with pollen on the first day of each instar and then provided with a mixture of pollen and soybean aphids until they had developed into the next instar. For adults, single pairs of *P. japonica* were fed with pollen or with a combination of pollen and soybean aphids every alternate day in the same Petri dishes. The pollen were directly sprinkled on the bottom of the dish, and the aphids were provided on 1-cm segments of heavily infested soybean seedlings. Pollen was replaced every other day and the aphids were replaced daily. In addition, an open 2-mL centrifuge tube containing solidified 1% agar solution was added to each dish as a water source. All of the food elements were provided *ad libitum*. For adults, several folded paper tapes (0.6 cm width, 10 cm length) served as oviposition substrates. Maize pollen was used in the first experiment, and rapeseed pollen was used in the second experiment as described in the following sections.

All experiments were conducted in a climatic chamber at 26 ± 1 °C, 75% ± 5% RH and a 16:8 h light: dark photoperiod.

### 4.5. Bt Maize Pollen Experiment

Using the feeding system described above, an experiment was conducted in which *P. japonica* were fed *Bt* or non-*Bt* (control) maize pollen. There were two treatments with 76 neonate *P. japonica* per treatment: (i) IE09S034 maize pollen containing Cry1Ie; and (ii) Z31 maize pollen (control). Larval survival, pupation rate, eclosion rate, and development time were recorded based on daily observations. When adults emerged (<12 h), they were individually weighted using an electronic balance (CPA224S; Sartorius AG; readability = 0.1 mg, repeatability ˂± 0.1 mg). Subsequently, the sex of the freshly emerged *P. japonica* adults was determined, and randomly selected pairs were continuously fed with *Bt* or non-*Bt* maize pollen and soybean aphids as described above. A total of 20–25 pairs of adult *P. japonica* were tested for each treatment. Survival and total fecundity (number of eggs laid per female) were recorded based on daily observation. This experiment was terminated after 19 days.

To determine the bioactivity of the *Bt* protein in the maize pollen during the experiment, three subsamples of *Bt* maize pollen and control pollen were taken before and after the two days feeding exposure. The samples were stored at −60 °C until further use.

### 4.6. Purified Cry Protein Experiment

The test system used for the purified Cry protein bioassay was the same as described by Zhang et al. [31]. Newly hatched larvae of *P. japonica* were tested for each of three treatment: (i) rapeseed pollen containing Cry1Ie protein at 200 μg/g dry weight (DW) of pollen; (ii) rapeseed pollen containing E-64 protein at 400 μg/g DW of pollen (positive control); and (iii) rapeseed pollen (negative control). The Cry1Ie protein concentration in the pollen diet was approximately 40 times that of the concentration in the Cry1Ie-transgenic maize pollen (5 μg/g fresh weight) [32]. E-64 served as a positive control since it is known to be (i) toxic to *P. japonica* at 400 μg/g DW, (ii) a gut-active compound like the Cry1Ie protein, and (iii) stable during the test duration [31,35]. Rapeseed pollen was used because it has been confirmed to be a highly nutritional food source for *P. japonica* and is commercially available. The pollen-based diets were prepared before the beginning of the experiment and stored at −20 °C until used. Soybean aphids were provided as a supplement dietary in each treatment as described above. Diets were replaced every two days to avoid the degradation of the test compounds.

The experiment was started with 82–84 neonate *P. japonica* per treatment. Larval development, survival, pupation rate, and eclosion rate were recorded based on daily observations. Adult *P. japonica* were weighted within 12 h of emergence. Subsequently, the sex of the freshly emerged adults was determined, and randomly selected pairs were individually kept in the dishes as described above. Thirty-one pairs of adults were test in the control treatment, and 30 pairs of adults were test in the Cry1Ie protein treatments, while only 21 pairs of adults were test in the E-64 protein treatments because of the low eclosion rates. The eggs laid by each female were recorded based on daily observation. After 20 days, the experiment was terminated.

To determine the bioactivity of *Bt* proteins in the rapeseed pollen-based diet during the experiment, three subsamples were taken from the pollen-based diet before and after it had been exposed to *P. japonica* for 2 days. The samples were stored at −60 °C until further use.

### 4.7. Sensitive-Insect Bioassay

The bioactivity of the Cry1Ie protein in *Bt* maize pollen and the rapeseed pollen-based diet before and after exposure to *P. japonica* for two days was determined with a sensitive-insect bioassay that used *C. suppressalis* larvae. 300 mg pollen and 4.6 g artificial diet of *C. suppressalis* larvae were weighted separately using an electronic balance. Subsequently, the pollen was thoroughly incorporated into the artificial diet for *C. suppressalis* larvae. The Z31 maize pollen and the control rapeseed pollen-based diet served as control treatments. The artificial diets were cut into slices and placed in Petri dishes (9 cm diameter, 1 cm height) with neonate larvae of *C. suppressalis* (one slice and one larvae per dish). Subsequently, the Petri dishes were sealed with Parafilm and reinforced with surgical tape. Each treatment was represented by 30 replicate dishes. After seven days, the *C. suppressalis* larvae were weighted.

### 4.8. Data Analyses

Pair-wise statistical comparisons were made between the *Bt* maize pollen and non-*Bt* pollen treatments in the first experiment, and between the Cry1Ie or E-64 treatments and the control in the second experiment. Chi-square tests were used to compare pupation rates and eclosion rates. Mann-Whitney *U*-tests were used to compare larval developmental times because such data did not satisfy the assumptions for parametric analyses (normal distribution of residuals and homogeneity of error variances).

Data on adult fresh weight and total fecundity were compared using Student’s *t*-test in the maize pollen experiment. Dunnett tests were conducted to compare adult weight and total fecundity in the Cry protein bioassay. In addition, Student’s *t*-test was carried out to compare the weights of *C. suppressalis* larvae that were fed with artificial diets containing different pollen treatments.

All statistical analyses were conducted using the software package SPSS (version22; SPSS, Inc., Chicago, IL, USA).

## Figures and Tables

**Table 1:** Effect of consumption of pollen from *Bt* maize (IE09S034) expressing Cry1Ie or the corresponding non-*Bt* maize (Z31) on life table parameters of *Propylea japonica*. Number of replicates is given in parentheses. For none of the parameters measured was a significant treatment effect detected.

Maize Line	Pupation Rate (%) ^a^	Eclosion Rate (%) ^a^	Days to Pupa (d ± SE) ^b^	Adult Fresh Weight (mg ± SE) ^c^		Total Fecundity per Pair (Eggs ± SE) ^c^
				Female	Male	
IE09S034	82.89 (76)	76.32 (76)	10.79 ± 0.20 (63)	6.48 ± 0.13 (29)	4.98 ± 0.18 (29)	80.64 ± 7.83 (25)
Z31	88.16 (76)	73.68 (76)	10.63 ± 0.17 (67)	6.41 ± 0.15 (34)	5.24 ± 0.15 (22)	93.40 ± 11.93 (20)

^a^ χ2 test; ^b^ Mann-Whitney *U*-test; ^c^ Student’s *t*-test.

**Table 2:** Effect of Cry1Ie protein or E-64 on different life table parameters of *Propylea japonica*. Larvae were fed a combination of rapeseed pollen, augmented or not with the insecticidal proteins, and soybean aphids. Number of replicates is given in parentheses.

Treatment	Pupation Rate (%) ^a^	Eclosion Rate (%) ^a^	Days to Pupa (d ± SE) ^b^	Adult Fresh Weight (mg ± SE) ^c^		Total Fecundity per Pair (Eggs ± SE) ^c^
				Female	Male	
Control: pure diet	85.71 (84)	78.57 (84)	8.69 ± 0.13 (72)	6.12 ± 0.11 (32)	5.05 ± 0.10 (34)	157.67 ± 13.19 (31)
Cry1Ie (200 µg/g diet)	87.80 (82)	84.15 (82)	8.81 ± 0.12 (72)	6.35 ± 0.14 (32)	5.20 ± 0.10 (37)	148.70 ± 13.70 (30)
E-64 (400 µg/g diet)	79.52 (83)	78.31 (83)	13.29 ± 0.18 (66)＊	5.28 ± 0.16 (26)＊	4.56 ± 0.09 (39)＊	22.40 ± 2.77 (21)＊

Each toxin treatment was compared to the control. An asterisk denotes a significant difference between a toxin treatment and the control; ^a^ χ^2^ test with Bonferroni correction (adjusted α = 0.025); ^b^ Mann-Whitney *U*-test with Bonferroni correction (adjusted α = 0.025); ^c^ Dunnett test. * An asterisk denotes a significant difference between a toxin treatment and the control.
